# Supplementary material for: Use of the Secreted Proteome of Trametes versicolor for Controlling the Cereal Pathogen Fusarium langsethiae
Source: Int J Mol Sci. 2019 Aug 26;20(17):4167. doi: 10.3390/ijms20174167 (PMC6747115; doi:10.3390/ijms20174167)
Supplement: Supplementary file 1 [file ijms-20-04167-s001.pdf]

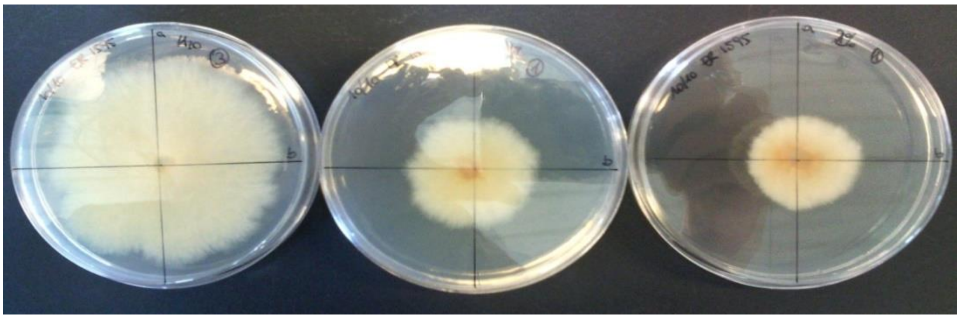

Figure S1. Growth inhibition assay of *F. langsethiae* (ER 1595) in presence of CF TV117. Petri dish non treated with CF on the left, in the middle and on the right Petri dishes treated with 0.04 % and 0.08 % (w/v) of CF TV117.

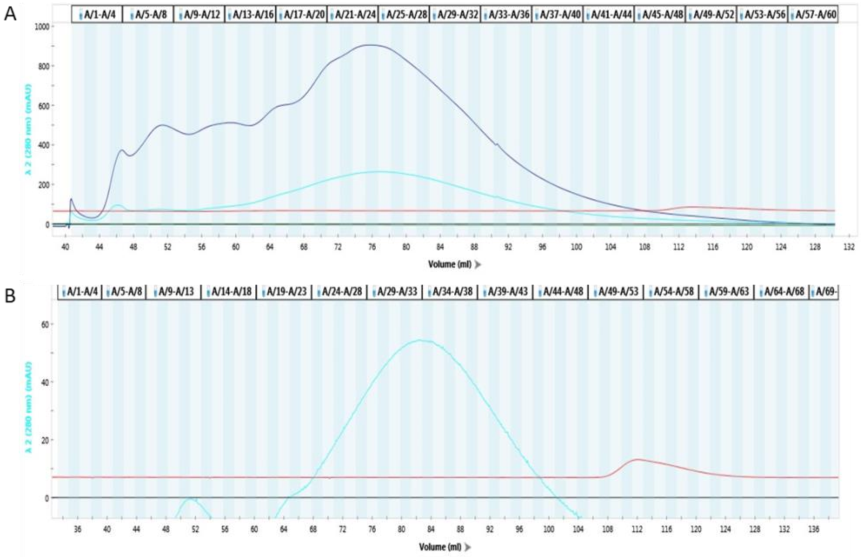

Figure S2: Sephacryl S-100 analysis of fractions : A, F75; B, F90.

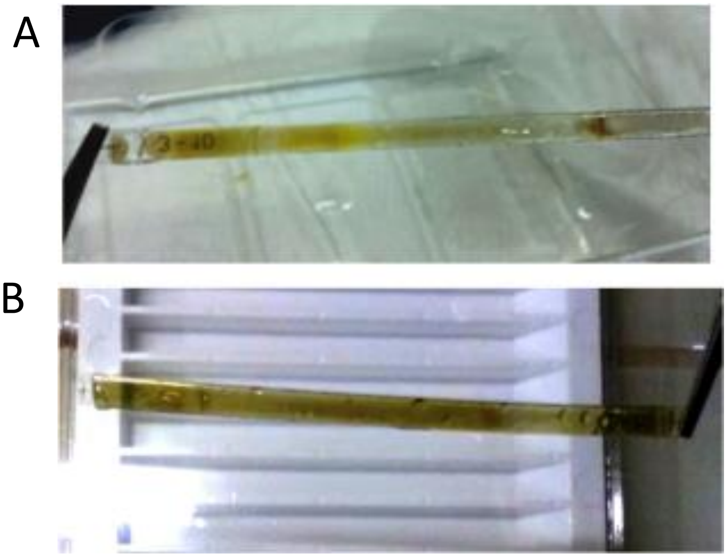

Figure S3: isoelectric focousing of the fractions F75_7 and F90_4, as example for determing the isoelectric point pI. The area with marked color indicates the regions where the proteins are positioned in the strip.

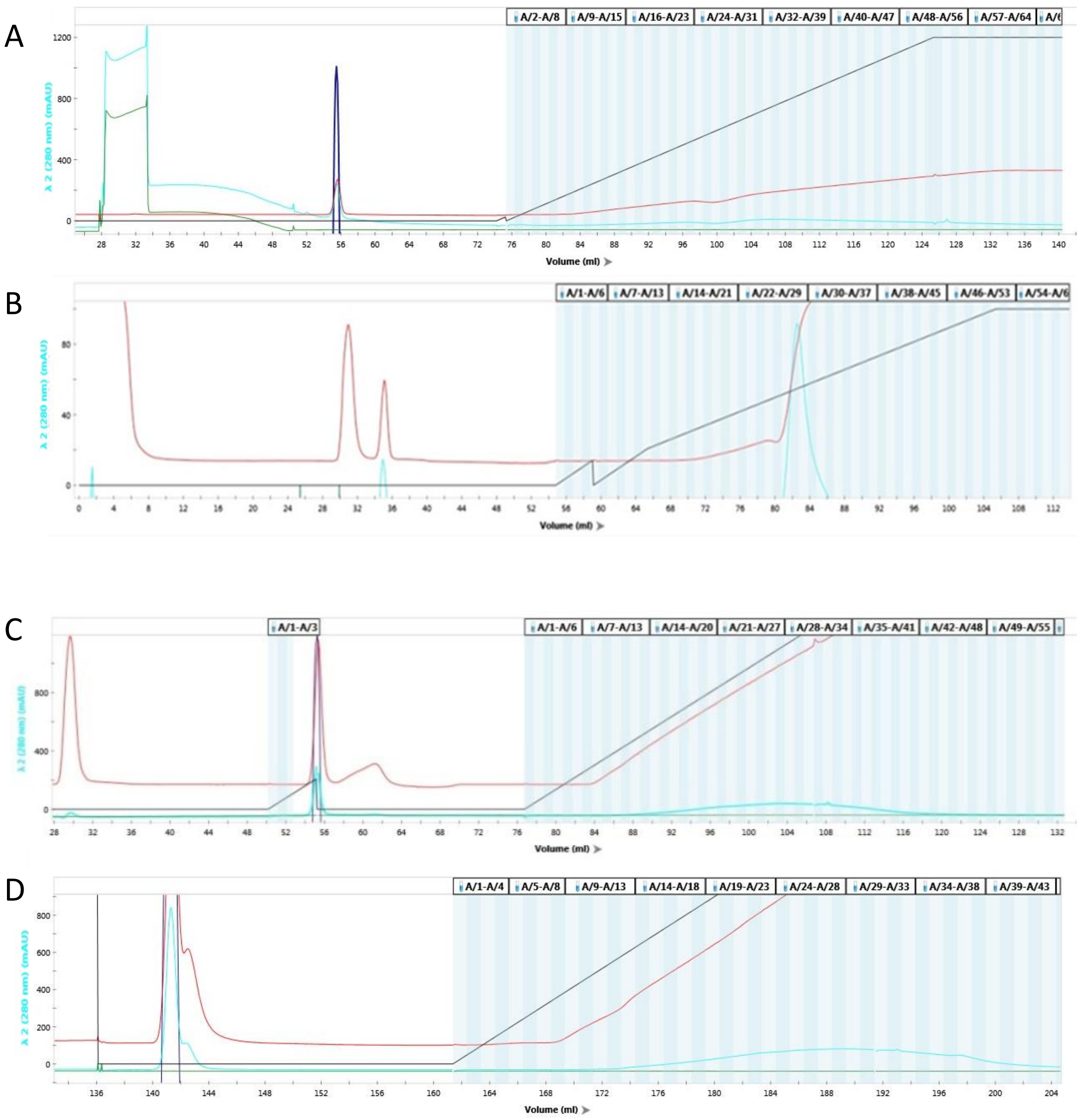

Figure S4: Chromatogram obtained from the HiTrap Q analysis of the fraction F75_7 (A), F90_2 (B), F90_4 (C), F90_5 (D).
